# *In vitro* Alternatives to Acute Inhalation Toxicity Studies in Animal Models—A Perspective

**DOI:** 10.3389/fbioe.2020.00549

**Published:** 2020-06-03

**Authors:** Dania Movia, Solene Bruni-Favier, Adriele Prina-Mello

**Affiliations:** ^1^Laboratory for Biological Characterisation of Advanced Materials (LBCAM), Department of Clinical Medicine, Trinity Translational Medicine Institute, Trinity College, The University of Dublin, Dublin, Ireland; ^2^AMBER Centre, CRANN Institute, Trinity College, The University of Dublin, Dublin, Ireland

**Keywords:** toxicity testing alternatives, inhalation studies, *In vitro* alternatives, air-liquid interface (ALI) culture, lung epithelium

## Abstract

When assessing the risk and hazard of a non-pharmaceutical compound, the first step is determining acute toxicity, including toxicity following inhalation. Inhalation is a major exposure route for humans, and the respiratory epithelium is the first tissue that inhaled substances directly interact with. Acute inhalation toxicity testing for regulatory purposes is currently performed only in rats and/or mice according to OECD TG403, TG436, and TG433 test guidelines. Such tests are biased by the differences in the respiratory tract architecture and function across species, making it difficult to draw conclusions on the potential hazard of inhaled compounds in humans. Research efforts have been therefore focused on developing alternative, human-relevant models, with emphasis on the creation of advanced *In vitro* models. To date, there is no *In vitro* model that has been accepted by regulatory agencies as a stand-alone replacement for inhalation toxicity testing in animals. Here, we provide a brief introduction to current OECD test guidelines for acute inhalation toxicity, the interspecies differences affecting the predictive value of such tests, and the current regulatory efforts to advance alternative approaches to animal-based inhalation toxicity studies. We then list the steps that should allow overcoming the current challenges in validating *In vitro* alternatives for the successful replacement of animal-based inhalation toxicity studies. These steps are inclusive and descriptive, and should be detailed when adopting in house-produced 3D cell models for inhalation tests. Hence, we provide a checklist of key parameters that should be reported in any future scientific publications for reproducibility and transparency.

## Introduction

Inhalation is a major exposure route for humans, where the respiratory tract serves as both target tissue and portal of entry (POE) to the systemic circulation for inhaled substances. REACH (Registration, Evaluation, Authorization, and Restriction of Chemicals) states that testing for acute inhalation toxicity is mandatory for all substances manufactured or imported at quantities above 10 tons per year when (i) human exposure is possible via this route or (ii) the physico-chemical properties of the substance indicate that such exposure may occur^1^. In this scenario, acute inhalation toxicity testing provides the data used for both hazard identification and risk assessment.

### Current Regulatory Test Guidelines for Acute Inhalation Toxicity

According to the Organization for Economic Co-operation and Development (OECD), acute inhalation toxicity testing is performed to define the effects of inhaled substances on (i) the respiratory tract (local toxicity) and/or (ii) the whole body (systemic toxicity) (OECD, 2018a).

Acute inhalation toxicity studies are currently conducted in animals by using the OECD methods TG403, TG436, and TG433. According to these methods, healthy young adult rats are the preferred animal model, and justification should be provided if other species are used (OECD, 2009a). Animals are exposed to the test compound as a gas, vapor, aerosol, or a mixture thereof. Nose-only exposure is generally recommended (OECD, 2009a). In special cases, whole-body exposure can be used, but this should be justified in the study report. Principles, advantage and disadvantages of the nose-only and whole-body exposure techniques are described in OECD Guidance Document 39 (OECD, 2018a). For both techniques, a single exposure is applied to each animal, with each exposure lasting up to 6 h in rats but not exceeding 4 h in mice. Animal observation is conducted for at least 14 days after exposure.

The endpoint of OECD TG403 and TG436 is “death.” A full description of these test guidelines is available on the OECD website (OECD, 2009b,c) and in Arts et al. (2008). In order to reduce the number of animals used and to improve their welfare (3Rs principle), an alternative fixed concentration procedure (FCP) was proposed in 2004 (draft OECD TG433^2^), where the endpoint “death” was replaced with “evident toxicity.” A previous study had demonstrated, in fact, that the performance of the FCP method in estimating the toxic class of inhaled substances was comparable to that of TG403 and TG436 tests (Stallard et al., 2003). However, the FCP test was dropped from the OECD workplan in 2007, due to suspected gender influences in the data generated, and the “subjective nature” of the tested endpoint. Conversely, few years later, scientists demonstrated that gender differences did not have any significant impact on the performance of the FCP test (Stallard et al., 2011). In parallel, an international working group that included 19 organizations around the world, led by the UK NC3Rs, developed the criteria to make “evident toxicity” into an objective and transferable endpoint. The working group carried out a large-scale analysis of inhalation toxicity data from 188 substances, and developed guidelines to support the recognition and use of “evident toxicity.” Such guidelines are described in Sewell et al. (2015). The scientific evidence provided by these two studies, supported the approval of the FCP method as OECD method in 2017 (OECD TG433). It should be noted here that, validation of the FCP method was critical at regulatory level for addressing the need for an OECD-approved inhalation toxicity test method that would satisfy the guidelines of Directive 2010/63/EU (EU, 2010). The latter states, *in vivo* testing methods should avoid, as far as possible, death as an endpoint, due to the severe suffering caused on the animal during the period prior to death.

### Interspecies Differences in the Respiratory Tract—How Does This Affect Acute Inhalation Toxicity Tests?

Species-specific differences can have important implications in acute inhalation toxicity testing, making it difficult to draw conclusions on the potential hazard of inhaled compounds in humans, for the reasons highlighted below.

When using animal models, two parameters are known to influence the local toxic effects of inhaled substances in the respiratory tract: (i) the pattern of deposition of the test substance, followed by (ii) the specific pathways by which the compound is cleared from the lungs. Animal models differ from humans in both aspects (Pauluhn, 2003). On one hand, the deposition of inhaled substances depends upon air-flow dynamics, which is different across species. Interspecies differences affecting air-flow dynamics include: the gross anatomy and geometry of airways in both the upper and lower respiratory tract (Parent, 2015), airway dimensions (e.g., length and diameter) (Hofmann et al., 1989), and respiratory physiology (e.g., breathing mode and ventilation rates). On the other hand, substance clearance is affected by tissue volumes, cell types and their location in the respiratory tract, mucus composition and distribution, macrophage-triggered clearance, biochemical mechanisms of airway activation, and enzyme-dependent metabolic processes. All the properties abovementioned are highly species dependent (Miller et al., 1993; Bogdanffy and Keller, 1999; Sarangapani et al., 2002).

In the following section, the specific differences between humans and the preferred animal model used in the three OECD accepted methods (rats) are briefly summarized.

#### Implications in the Use of Rodents in the OECD Tests

In the last two decades scientists have demonstrated that the relevance of using rats (the preferred animal model in OECD tests) for assessing hazard and risk of chemicals in humans, is scientifically debatable (Harkema, 1991; Mauderly, 1997; Phalen et al., 2008; Creton et al., 2010; Chamanza and Wright, 2015; Mowat et al., 2017). For example, recently, a review of 52 inhalation toxicity studies conducted in rodents, showed that the results obtained from such studies lack relevance to humans (Mowat et al., 2017),

Probably the most obvious and significant difference between humans and rodents is the anatomy of their lungs. Rat lungs have a monopodial branching system with no respiratory bronchioles; whereas, the human respiratory tract has a symmetric branching. This results in compound/particle deposition mainly at the bifurcation points of the human lungs, a phenomenon that cannot be mimicked in rodent models. Also, rat airway diameter is smaller than human one. Thus, insoluble solid aerosols can lead to an obstruction of the rat airways and, subsequently, to animal death, at the highest tested doses, even when the compound under investigation is non-toxic to humans (Hofmann et al., 2018).

Furthermore, the breathing mode of humans is different from rodents. Humans are oronasal breathers, while rodents are obligate nose breathers. This strongly influence how inhaled particle and gas deposit in the respiratory tract, and the subsequent toxicities detected. For example, there is less filtering of particles and gases in oral breathing compared to nasal breathing, resulting in a greater delivery of material to the peripheral airways of humans compared to rodents.

Differences in compound metabolism are also striking (Bogdanffy and Keller, 1999; Sarangapani et al., 2002; Oesch et al., 2019). Cytochrome P450 in the nasal mucosa and lower respiratory tract of humans is poorly efficient, as compared to that of most animal species, including mice and rats. Clearance via carboxylesterase activity is also particularly ineffective in humans as compared to rodents. On the other hand, phase II enzymes (e.g., epoxide hydrolase and glutathione S-transferase) are more active in humans than in rodents, enabling a clearance of inhaled compounds that cannot be replicated in the rat/mice models used in the OECD tests.

Finally, reflex reactions that are of a protective nature in rodents, can limit the animal exposure to the chemicals under investigation. Reflex reactions range from mechanisms where the animal use its own fur as a filter to aerosol exposure, causing dosimetry issues in whole-body exposure techniques (reviewed in Pauluhn, 2003), to the stimulation of the parasympathetic system, resulting in reactions that can be confused with early toxic effects, such as decrease in ventilation rate, heartbeat, blood pressure, and body temperature of the animal.

The OECD is aware of the interspecies differences listed above and their negative influence on the predictivity of the existing inhalation toxicity tests. Subsequently, a new test for determining acute inhalation toxicity has been recently brought forward for validation and OECD adoption (Jackson et al., 2018). Such test adopts the EpiAirway™ model, a ready-to-use, three-dimensional (3D) *In vitro* mucociliary tissue model consisting of normal, human-derived tracheal/bronchial epithelial cells cultured at the Air-Liquid Interface (ALI). The test under development could, therefore, provide a human-relevant *In vitro* alternative to current, animal-based acute inhalation toxicity studies.

### *In vitro* Alternatives to Acute Inhalation Toxicity Studies in Animals

Considering the limitations of animal models in predicting the safety of inhaled substances in humans, research efforts have focused on developing human-relevant models, with particular emphasis on *In vitro* models, such as the EpiAirway™ model mentioned above. Our perspective focuses on the steps that should allow these models to increase the predictive value of acute inhalation toxicity testing, by overcoming some of the shortfalls of animal models. Indeed, inadequate physico-chemical characterization of the test compound and dosimetry can also lead to unpredictive results in inhalation toxicity tests. However, our manuscript does not address issues associated with exposure technology, test compound characterization and dosimetry, as these have already been identified and described in detail elsewhere (Dorato and Wolff, 1991; Oberdorster, 1996; Pauluhn, 2003, 2005; Wong, 2007; Phalen and Mendez, 2009; Clippinger et al., 2018b; Hofmann et al., 2018).

Human-relevant *In vitro* models allow reproducing distinctive properties and mechanisms of the human lung epithelium that define the clearance of inhaled compounds in humans. The properties/mechanisms reproduced include tissue volumes, cell and mucus composition, human-specific aspects of macrophage-triggered clearance, and unique human biochemical and enzyme-dependent processes. To date, the most advanced *In vitro* approaches for animal replacement detect local toxicity. Thus, this manuscript focuses solely on such endpoint.

Numerous reviews on *In vitro* inhalation toxicity testing models have been published in the last decade (Berube et al., 2009; Creton et al., 2010; Gordon et al., 2015; Clippinger et al., 2018b; Lacroix et al., 2018; Upadhyay and Palmberg, 2018). These models can be grouped in three main categories: (i) cell cultures, including commercially available, 3D *In vitro* lung models; (ii) lung-on-a-chip models, and (iii) *ex vivo* human precision-cut lung slices. Various case studies demonstrate that it is possible to reproduce specific regions of the human respiratory tract and their responses *In vitro*. For example, in 2018, the US Environmental Protection Agency (EPA) has publicly recognized the value of an alternative approach based on an *In vitro* model of the human lung epithelium (the MucilAir™ model), to refine inhalation risk assessment for the pesticide chlorothalonil, as well as for other contact irritants (EPA, 2018). However, currently there is no *In vitro* model that has been accepted by regulatory agencies as a stand-alone replacement for animal tests in acute inhalation toxicity studies, and the issues associated with interspecies differences remain unsolved.

#### Regulatory Efforts to Advance Alternative Approaches to Animal-Based Inhalation Toxicity Studies

In the last years, regulatory efforts have been focused on advancing the alternative approaches for replacing animal use in acute inhalation toxicity testing, as reported by Clippinger et al. (2018b) and Krewski et al. (2020). For example, an Interagency Coordinating Committee on the Validation of Alternative Methods (ICCVAM)^3^ has been formally established in US in 2000, with the aim of (i) evaluating existing *in vivo, in silico*, and *In vitro* tests for acute systemic toxicity, and (ii) developing a strategic roadmap^4^ where *In vitro* and *in silico* approaches enable the reduction, or the full replacement, of current *in vivo* tests. Similarly, the Office of Pesticide Programs (OPP) of EPA has committed to significantly reduce the number of animals used for acute inhalation toxicity testing in the agrochemical registration process (EPA, 2016)^5^. EPA has also announced that funding to studies in mammals will be ended by 2035.

In Europe, in 2016, the Netherlands National Committee for the protection of animals used for scientific purposes (NCad) announced that animal studies for safety research on chemical substances, food ingredients, pesticides, and medicines (including veterinary medicines) will be phased out in the Netherlands by 2025 (NCad, 2016). This ambitious objective is backed up also by the European Commission. In 2005, the European Partnership for Alternative Approaches to Animal Testing (EPAA) was established, with the aim to replace, reduce and refine (3Rs concept) animal use in regulatory testing. Furthermore, Directive 2010/63/EU (EU, 2010) explicitly incorporates the 3Rs concept in European legislation, and establishes the European Union Reference Laboratory—European Centre for the Validation of Alternative Methods (EURL—ECVAM) at the Joint Research Centre (JRC) as support to the development, validation, and acceptance of alternative methods. The European Commission is also currently funding several research projects in the alternatives field (e.g., EU-ToxRisk).

Globally, the International Cooperation on Alternative Test Methods (ICATM) was established. ICATM includes governmental organizations from Europe, US, Canada, Japan, South Korea, Brazil, and China.

Although all initiatives above create a momentum toward the replacement of animal testing, the translational rate of *In vitro* alternatives into regulator-approved methods is poor. Thus, one could question the predictive value of such alternatives. The reality is, validation of *In vitro* testing methods for animal replacement is currently a gray area (Griesinger et al., 2016). Regulatory authorities grant validation to *In vitro* alternative tests upon demonstration of their ability to predict *in vivo* animal-derived data, the quality and reliability of which is sometimes poor (Sauer et al., 2013). The scientific relevance of using animal data as benchmark for a human-relevant model is also debatable (Griesinger et al., 2016; Cryan et al., 2019), since the interspecies differences described in the previous sections negatively affect the animal-to-human data correlation. In Europe, the mandatory steps for validation are: (i) endorsement from the European validation authority, i.e., the EURL—ECVAM; (ii) formal test methods via large international collaboration platforms, such as the OECD or the International Council on Harmonization; (iii) regulatory acceptance; and (iv) deletion of the animal test. Thus, the current validation process is tremendously demanding, taking an average of 10 years and costing up to 1 million dollars (Hartung, 2013). This approach raises the bar to an unaffordable level for small technology providers and universities, creating a translational “valley of death.” Predictive, human-relevant *In vitro* platforms are published in peer-reviewed scientific journals, but do not go through the validation process.

In this context, the following section presents the authors' perspective on how, in our view, it may be possible to overcome the current challenges in validating *In vitro* alternatives for the successful replacement of animal-based inhalation toxicity testing studies.

## Discussion

Although in some cases *In vitro* alternative tests are at an advanced stage of development (e.g., the EpiAirway™ model mentioned above), to date all *In vitro* alternative models for inhalation toxicity studies still fall into the category of “non-guideline methods.” Four major actions should be undertaken, in our view, to increase the uptake of *In vitro* alternative methods and meet the replacement of animal models for the definition of local toxicity in acute inhalation testing.

Firstly, *In vitro* test methods should be presented in detail to allow interpretation and use of the data from regulators. According to regulatory agencies, non-guideline methods can be used to support risk/hazard assessment of inhaled substances only if they fulfill basic requirements, such as relevance, reproducibility and predictivity. The OECD has recently formulated a guidance document (GD211) on the information that should be provided for non-guideline methods. Further to this, an annotated toxicity test method template (ToxTemp), described in full by Krebs et al. (2019, 2020), was developed, to complement the OECD GD211 guidance document and support researchers in meeting its requirements. Furthermore, the test methods and conditions under which the data are generated, must adhere to the Good *In vitro* Method Practices (GIVIMP) for the development and implementation of *In vitro* methods for regulatory use in human safety assessment (OECD, 2018b). We believe that the mandatory adoption of the ToxTemp template and GIVIMP procedures by the scientific community, will facilitate implementation of *In vitro* alternative methods for inhalation toxicity testing.

**Figure 1:**
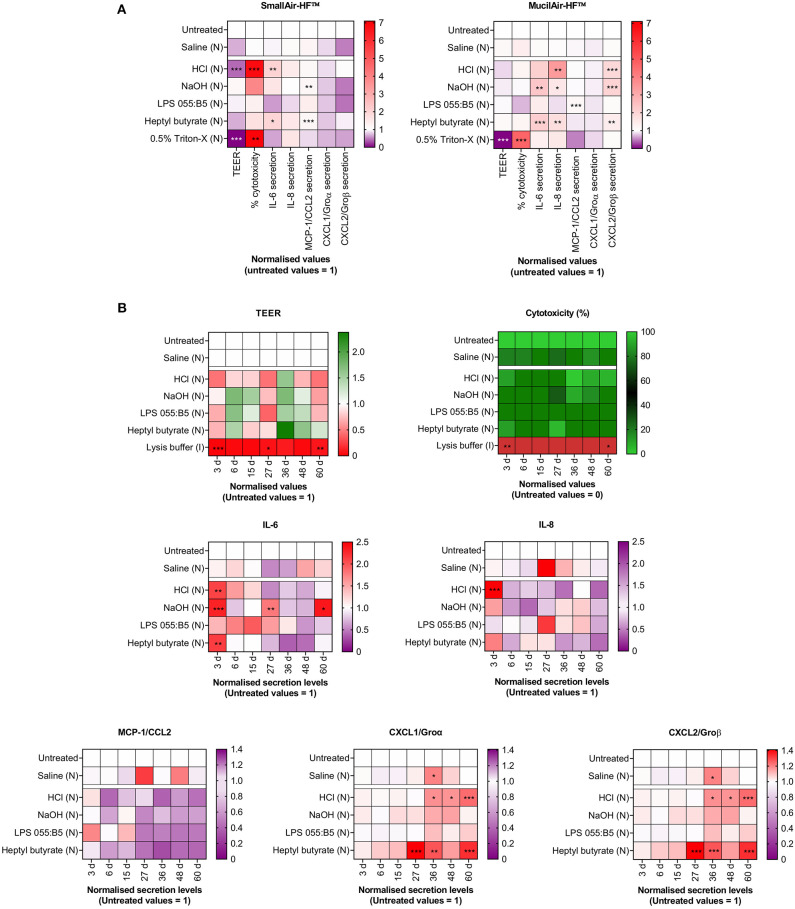
Changes in markers of cellular- and tissue-specific KEs following a single-dose aerosol (N) exposure to benchmark substances. Cell cultures were exposed to liquid aerosols by means of a Vitrocell Cloud ALI system equipped with an Aeroneb® Pro nebulizer. Cellular-specific KEs included percentage (%) cytotoxicity, cytokines (IL-6, IL-8) and chemokines (MCP-1/CCL2, CXCL1/Groα, CXCL2/Groβ) secretion. Tissue-specific KEs included epithelial barrier impairment, quantified as changes in TEER. Data are presented as mean and normalized to untreated cultures. **(A)** SmallAir-HF™ (left) and MucilAir-HF™ (right) models were exposed to benchmarks for 72 h. **(B)** MucilAir-HF™ models were exposed to benchmarks up to 60 days. **(A,B)** Symbols (*), (**), and (***) indicate *p* < 0.05, *p* < 0.01, and *p* < 0.001, respectively (two-way ANOVA followed by Dunnett post-test; comparison to the untreated controls).

Secondly, *In vitro* alternatives must be compatible with the evaluation of markers of membrane/cell damage and cell functional competence that are relevant to known adverse outcome pathways (AOPs). As recently reviewed by Clippinger et al. (2018a), AOPs can model the mechanisms leading to adverse local and systemic effects following compound inhalation. By using cellular- and tissue-specific Key Events (KE) reported in inhalation AOPs as experimental endpoints, the authors have successfully investigated the predictive value of reconstructed human lung tissue cultures in detecting POE inflammatory effects. The results of such investigation (the details of which are described in the Supplementary Material) are shown in Figure 1 and are original and unpublished data from the authors. Comparable experimental strategies have been successfully adopted by other research groups to validate the predictive value of *In vitro* alternative models (Iskandar et al., 2017; Balogh Sivars et al., 2018; Hoffmann et al., 2018; Barosova et al., 2020). In our experiments, SmallAir-HF™ and MucilAir-HF™, purchased from Epithelix Sárl, were used (Supplementary Figure S1). Experimental endpoints included changes in: (i) percentage cytotoxicity and release of cytokines (IL-6, IL-8)/chemokines (MCP-1/CCL2, CXCL1/Groα, CXCL2/Groβ), as markers of cellular-specific KEs; and (ii) trans-epithelial electrical resistance (TEER) as marker of a tissue-specific KE (the epithelial barrier integrity). Marker expression was evaluated after a single aerosol exposure to benchmark substances with known effects on the respiratory epithelium in humans. These included: (i) hypertonic saline solution, a biocompatible nebulization vehicle, as negative control; (ii) chemical lung irritants (hydrochloric acid, HCl, and ammonium hydroxide, NaOH); (iii) lipopolysaccharide (LPS) from *E. Coli* 055:B5, a biological contaminant to which the respiratory system is directly exposed, and that does not cause irritation unless the epithelial barrier function is impaired, such as, for example, in the presence of pre-existing medical conditions (e.g., asthma, cystic fibrosis); (iv) heptyl butyrate, which is known to be non-irritant if inhaled at doses lower than 200 mg/ml; and (v) 0.5% Triton-X or lysis buffer, which are cytotoxic compounds, as positive controls. After 72 h exposure (Figure 1A), benchmark compounds induced the predicted response. Saline did not induce any significant change in TEER and did not trigger cytotoxicity. Similar results were found after exposure to LPS and the non-irritant heptyl butyrate. Triton-X significantly disrupted the barrier integrity (TEER ~ 0) and caused severe cytotoxicity. Lung irritants (HCl and NaOH) caused barrier integrity disruption, cytotoxicity and/or release of inflammatory signals. The unaltered viability of untreated MucilAir-HF™ cultures after 60 days (Figure 1B), together with the evident time-dependent inflammatory responses detected for the benchmarks in the same time period, suggests that the here presented *In vitro* model could be used to carry out long-term experiments as an alternative method to acute inhalation toxicity studies in animals.

**Figure 2:**
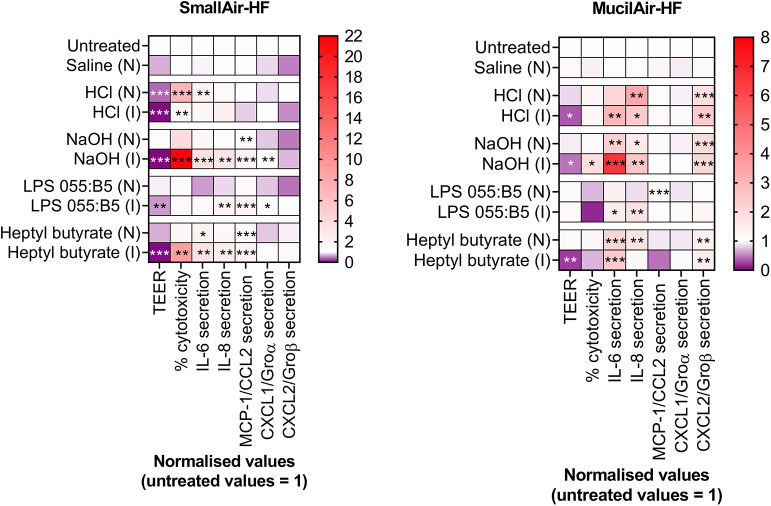
Changes in markers of cellular- and tissue-specific KEs in SmallAir-HF™ (left) and MucilAir-HF™ (right) models following a single-dose exposure by aerosol (N) or by direct inoculation (I). Cellular-specific KE included percentage (%) cytotoxicity, cytokines (IL-6, IL-8) and chemokines (MCP-1/CCL2, CXCL1/Groα, CXCL2/Groβ) secretion. Tissue-specific KE included epithelial barrier impairment, quantified as changes in TEER. Data are presented as mean and normalized to untreated cultures. Symbols (*), (**), and (***) indicate *p* < 0.05, *p* < 0.01, and *p* < 0.001, respectively (two-way ANOVA followed by Dunnett post-test; comparison to the untreated controls).

Thirdly, *In vitro* acute inhalation toxicity testing should use exclusively cell cultures in ALI conditions, i.e., cultures where cells are grown in direct contact with air. ALI culturing conditions are in fact a critical element driving the *In vitro* formation of a pseudostratified epithelium that mimics the human lung epithelium functions in the best possible way (Gras et al., 2017; Hiemstra et al., 2018). Furthermore, ALI cultures should be exposed to the test compound in realistic exposure conditions by means of realistic exposure techniques (e.g., gas, vapor, aerosol). The influence of exposure methods on cell responses in ALI cultures has been described in the past by the authors (Di Cristo et al., 2018; Movia et al., 2018). This was also supported by further experiments we recently carried out on MucilAir-HF™ and SmallAir-HF™, showing that inoculation (I) or nebulization (N) of the same compound on the apical side of the two models triggered cellular responses that significantly differed (Figure 2). Finally, exposure should be clearly characterized, including among the parameters particle size distribution, nominal and actual/deposited concentrations, as described in the OECD guidelines for testing acute inhalation toxicity (OECD, 2009a).

Fourthly, the adoption of ready-to-use ALI models of the human respiratory epithelium, reconstituted from biopsies originated from human donors, should be preferred to in-house *In vitro* systems based on immortalized cell lines. Ready-to-use systems, which are available for purchase from commercial sources (e.g., EpiAirway™, MucilAir-HF™, SmallAir-HF™), offer in fact a standardized platform, thus facilitating method validation and promoting consistency across laboratories. Furthermore, selecting donors of different age and gender, ensures that hazard/risk assessment studies address the issues of population heterogeneity and gender dimension. However, the high cost of ready-to-use systems often hinders their use in research and university labs. Within the EC scenario of the H2020-funded BIORIMA and REFINE projects, which have enabled regulatory-science dialogue and experimental evidence discussion, we have highlighted that, when using in house-produced *In vitro* models for conducting inhalation toxicity studies, it is of critical importance to report the methodologies used for the formation, characterization, exposure, verification, validation and testing of the ALI cell model. On this matter, in Table 1, we propose a checklist describing key parameters that, in our opinion, should be included in future scientific publications when adopting in house-produced ALI cell models to test inhaled substances. This information should be also submitted as part of section 3 of the ToxTemp document, thus ensuring reproducibility, repeatability, and a transparent assessment of the predictive value of the *In vitro* method developed. This will ultimately allow a meaningful comparison between novel, animal-free, non-guideline methods and current OECD tests.

**Table 1:** Checklist describing key parameters that should be included in scientific publications, as well as in Section 3 of the ToxTemp document, when adopting in house-produced 3D cell models to test inhaled substances.

**Checklist descriptor**	**3D cell culture parameter**	**Information to be provided**
Culturing substrate	Scaffold-based	Scaffold material and structure
	Scaffold-free	Specialized cell culture plates or lab equipment (e.g. 3D printing) used
Cells	Cell types	Mono- or co-culture, primary cells, immortalized and/or carcinogenic cell lines, differentiation protocol
	Donors	Gender-balanced pool of cell donors
Cell culture formation	Methodology	Air-Liquid Interface (ALI) conditions, cell seeding on scaffolds, incorporation into matrices, liquid overlay, partially separated or mixed ALI co-cultures
	Growth time	Number of days/weeks
Cell culture manipulation	Biological cues	Medium change, mechanical cues (e.g. substrate stiffness, sheer flow), soluble/chemical cues (e.g. hormones)
Biological functions of cells	Cell phenotype	Cell shape, polarity, proliferative activity, cell differentiation
Biological functions of culture	Geometry	Culture morphology (2D or 3D) and architecture, culture size.
	Stability	Viability and phenotype changes overtime
	Comparison to tissue in humans	Cell-ECM and cell-cell interactions, formation of tissue-mimetic structures, mucus production
Exposure	Exposure methodologies	Human-relevance of experimental exposure conditions
Verification	Model benchmarking	Comparison to known, human-relevant exposure scenarios
Validation	Benchmarks	Benchmark identification and validation of cell culture responses
Assay validation for toxicity/efficacy testing	Endpoints	Human-relevant endpoint definition (e.g. based on AOPs), overcoming diffusion issues (e.g. during immunostaining), positive controls
	Accuracy	Benchmark data should include information on the variability and the upper and lower limits of accuracy metrics, as suggested by Leontaridou et al. (2019)

In addressing the four points above, consideration should be also given to the respiratory tract region/s where the inhaled substance under test is more likely to deposit in humans, followed by development of an *In vitro* model that is representative of such region/s. *In vitro* models representative of different respiratory tract regions, in fact, respond differently to the same irritant insult. Our results demonstrate that the MucilAir-HF™ model was less prone than the SmallAir-HF™ culture to develop inflammatory responses following exposure to the chemical irritants HCl and NaOH (Figure 1A). Furthermore, to avoid any uncertainty introduced by the interspecies differences associated with the use of animal products, *In vitro* alternatives should be fully humanized. Thus, only human cells should be used, and fetal bovine serum (FBS) and animal-derived ECM proteins should be avoided, as clearly addressed in Jochems et al. (2002), Gstraunthaler (2003), Van Der Valk et al. (2004, 2018), OECD (2018b), and Oredsson et al. (2019). Cell-to-cell ratios of the human tissues, as well as the endogenous lung microbiome, should also be recapitulated.

As highlighted by the recommendations from the 2015 workshop entitled “Alternative Approaches for Identifying Acute Systemic Toxicity: Moving from Research to Regulatory Testing,” we share the view that there is still need to improve the *In vitro* models for completely replacing animal use in acute inhalation toxicity testing (Hamm et al., 2017). Based on our experience in the development of advanced ALI cultures (Movia et al., 2017, 2018, 2019; Di Cristo et al., 2018), we have identified two critical shortfalls in the ALI culture models currently commercially available or reported in the scientific literature. They do not incorporate either (i) the 3D tissue microenvironment, constituted by different cell types, in direct contact with each other, and the extracellular matrix (ECM); or (ii) the tissue biomechanical environment (namely, the epithelial stretching during breathing). These two parameters strongly influence local inhalation toxicity, which is mainly affected by the nature of the interactions between the inhaled substance itself and the surrounding biological environment. To overcome these shortfalls, we suggest that future research efforts would focus on developing advanced ALI cultures formed by mixed cell populations that exist on ECM-like, 3D synthetic hydrogels. Furthermore, *In vitro* ALI models should undergo cyclic mechanical strains, mimicking the forces exerted during breathing, as these have been demonstrated to correlate to the absorption of inhaled substances (Huh et al., 2010) and to the epithelium inflammatory responses (Rentzsch et al., 2017).

In conclusion, we strongly advocate for enforcing standardization within the development of *In vitro* models for inhalation toxicity testing, and the uptake of the checklist in Table 1 within the ToxTemp framework. This will enable reproducibility and repeatability in this field, ensuring a rapid uptake of alternative methods from the regulatory agencies. It will also ensure the production of valuable data for *in silico* PBPK modeling, further supporting animal replacement in acute inhalation toxicity testing.

## Data Availability Statement

The datasets generated for this study are available on request to the corresponding author.

## Author's Note

The views expressed in this article are those of the authors. Mention of trade name or commercial products does not constitute endorsement or recommendation for use.

## Author Contributions

DM and AP-M conceived this study. DM designed and carried out the experimental work, with exception of ELISA experiments, which were carried out by SB-F. DM analyzed the data and discussed them with AP-M. DM drafted the paper, and AP-M revised it. All authors read and approved the final manuscript.

## Conflict of Interest

The authors declare that the research was conducted in the absence of any commercial or financial relationships that could be construed as a potential conflict of interest. The views expressed in this article are those of the authors. Mention of trade names or commercial products does not constitute endorsement or recommendation for use.

## Footnotes

^1^REACH. *Annex VIII: Standard Information Requirements for Substances Manufactured or Imported in Quantities of 10 Tonnes or More*. Available online at: https://reachonline.eu/reach/en/annex-viii.html (accessed April 2020).

^2^Available online at: http://www.oecd.org/chemicalsafety/testing/32035886.pdf (last accessed April 2020).

^3^Available online at: https://ntp.niehs.nih.gov/whatwestudy/niceatm/iccvam/iccvam-agencies/index.html (accessed April 2020).

^4^Available online at: https://ntp.niehs.nih.gov/whatwestudy/niceatm/natl-strategy/index.html?utm_source=direct&utm_medium=prod&utm_campaign=ntpgolinks&utm_term=natl-strategy (accessed April 2020).

^5^Available online at: https://www.epa.gov/pesticide-registration/mixtures-equation-pilot-program-reduce-animal-testing (accessed April 2020).

## Supplementary Material

Details on the experimental methods used to generate the data presented in the manuscript are reported in the Supplementary Information. Supplementary Figure S1 is also reported within the Supplementary Information file.
