# Supplementary material for: In vitro Alternatives to Acute Inhalation Toxicity Studies in Animal Models—A Perspective
Source: Front Bioeng Biotechnol. 2020 Jun 3;8:549. doi: 10.3389/fbioe.2020.00549 (PMC7284111; doi:10.3389/fbioe.2020.00549)
Supplement: Supplementary file 1 [file Data_Sheet_1.DOCX]

*In Vitro* Alternatives to Acute Inhalation Toxicity Studies in Animal Models – A Perspective

Dania Movia^1*^, Solene Bruni-Favier^1^, Adriele Prina-Mello^1,2^

^1^LBCAM, Trinity Translational Medicine Institute, Department of Clinical Medicine, Trinity College The University of Dublin, Dublin, Ireland

^2^AMBER Centre, CRANN Institute, Trinity College The University of Dublin, Dublin, Ireland

*** Correspondence:**Dr Dania Movia
dmovia@tcd.ie

Supplementary Material

# Supplementary Experimental Methods

This section describes the experimental methods used to generate the data presented within the main manuscript and used to support the authors’ perspective.

## MucilAir-HF™ and SmallAir-HF™ models

MucilAir-HF™ and SmallAir-HF™ cultures were used for the experiments described within the manuscript, and were purchased from Epithelix Sarl (Geneva, Switzerland). MucilAir-HF™ and SmallAir-HF™ cultures are 3D human airway epithelia reconstituted *in vitro* from donor biopsies. They are formed by a pseudostratified columnar epithelium of primary epithelial cells, presenting beating cilia and mucus production, co-cultured with human airway fibroblasts (HF) (Figure S1). The two models are representative of two different anatomical sites: large bronchial airways for MucilAir-HF™, and small airways for SmallAir-HF™. A pool of healthy human donors, of different age and gender, were selected (Figure S1), thus ensuring that our study addressed the issues of population heterogeneity and gender dimension in risk assessment research.

MucilAir-HF™ and SmallAir-HF™ cultures were supplied on Corning Costar Transwell™ Permeable Supports with PET membrane inserts of 6.5 mm of diameter and pore size of 0.4 μm. They were cultured in ALI conditions in 24-well plates placed in an incubator (37°C; 5% CO_2_) using a specific FBS-free culture medium (MucilAir™ Culture Medium and SmallAir™ Culture Medium), also purchased from Epithelix Sarl. Cell models were cultured for at least a week following arrival at the lab premises and prior to carrying out any experiment, to allow full cell recovery post transportation. TEER was also assessed as quality control; a TEER ≥ 200 Ωcm^2^ was considered adequate.

**Supplementary Figure S1.** Schematic representation of the composition of (A) MucilAir-HF™ and (B) SmallAir-HF™ cultures, and list of the biopsy donors selected for this study as reported in the supplier’s certificate of analysis.

## *In vitro* exposure to benchmark substances

### Benchmark substances – Compounds and concentrations

Benchmarks were purchased from Sigma-Aldrich (Dublin, Ireland) and diluted at the tested concentration in hypertonic saline. The latter was composed by 0.9% sodium chloride (NaCl) solution (w/v) suitable for cell culture, supplemented with 1.25 mM calcium chloride (CaCl_2_) and 10 mM N-2-hydroxyethylpiperazine-N-2-ethane sulfonic acid (HEPES) (all purchased from Sigma-Aldrich), as described in previous publications of some of the authors (Movia et al., 2017;Di Cristo et al., 2018;Movia et al., 2018;Movia et al., 2019). Benchmarks included: hydrochloric acid (HCl; 50 mM); ammonium hydroxide (NaOH; 75 mM); heptyl butyrate (200 mg/ml); and lipopolysaccharide (LPS) from *E. Coli* 055:B5 (100 ng/ml). Untreated cultures and models exposed to hypertonic saline were used as negative controls. Triton X (0.5%) or lysis buffer were used to induce cell death, as positive control.

### Exposure methods

Prior to exposure, the culture apical side was washed to remove the excess mucus produced by the epithelial cells overtime, according to the supplier’s recommendations. The apical side of the MucilAir-HF™ and SmallAir-HF™ cultures was then exposed to benchmarks and controls via direct inoculation with a pipette (I) or via aerosol (N). Pipette inoculation (I) was carried out as previously described by the authors (Di Cristo et al., 2018;Movia et al., 2018;Movia et al., 2019). Aerosol exposure (N) was carried out by means of Vitrocell Cloud ALI system (Vitrocell, Germany). This system is specifically designed for dose-controlled and spatially-uniform deposition of liquid aerosols on cells cultured in ALI conditions. The system is equipped with an Aeroneb® Pro nebulizer, a small-volume nebulizer based on vibrating-mesh technology (Aerogen Ltd., Galway, Ireland), controlled by an Aerogen-Pro X controller. It should be noted that this nebulizer is approved for clinical settings, and its use is widespread in hospitals. According to the manufacturer characterization by the Anderson Cascade Impactor technique, the mass median aerodynamic diameter (MMAD) of the aerosol droplets produced by our nebulizer head ranged between 4.0 and 6.0 ± μm. The nebulizer output rate was validated before each experiment, and was equal to 0.53 ± 0.07 ml/min. The aerosol was applied for a short time of approximately 1.5-2 minutes, allowing for the nebulization of 1 ml of benchmark/control solution.

## Evaluation of markers of Key Events (KEs) in acute inhalation toxicity

KEs were investigated at 72 h after exposure in SmallAir-HF™ cultures, and up to 60 days after exposure in MucilAir-HF™ models. Supernatants in the basolateral compartment of the ALI cultures were harvested at the time-point under investigation. Each benchmark and time-point were tested in duplicate (n_replicates_ = 2), and experiments were repeated three times (n_tests_ = 3). Data are presented in heatmaps created by Graph-Pad Prism 8 (Graph-Pad Software Inc., La Jolla, CA, USA) based on the data mean.

### Markers of cellular-specific KEs

#### Cytotoxicity

The percentage (%) cytotoxicity was quantified as lactate dehydrogenase (LDH) leakage by Thermo Scientific Pierce LDH Cytotoxicity Assay Kit (Fisher Scientific, Ireland), following the procedure previously described by the authors (Movia et al., 2018).

#### Cytokines secretion

The production of pro-inflammatory cytokines (IL-6 and IL-8) and chemokines (MCP-1/CCL2, CXCL1/Groα, CXCL2/Groβ) was evaluated by quantifying their concentration in the medium isolated from the basolateral compartment of the MucilAir-HF™ and SmallAir-HF™ models. The following commercially available sandwich Enzyme-Linked ImmunoSorbent Assay (ELISA) kits were used: Human IL-6 ELISA MAX™ Standard Set, Human IL-8 ELISA MAX Standard Set, Human MCP-1/CCL2 ELISA MAX™ Deluxe Set (all from BioLegend, MSC, Dublin, Ireland); Human CXCL1/GROalpha DuoSet ELISA and Human CXCL2/GRObeta DuoSet ELISA (R&D Systems, BioTechne, UK). ELISAs were carried out according to the manufacturers’ protocol and each sample was tested in duplicate. The Epoch microplate reader (Biotek, Mason Technology Ltd, Dublin, Ireland) was used to detect the optical density at 450 nm for each well, and the determined values were corrected by subtracting the optical aberration of the 96-well plastic plate at 570 nm. The means of the resulted values were calculated and calibrated against a standard curve.

### Markers of tissue-specific KEs

#### Trans-epithelial electrical resistance (TEER)

TEER measurements were carried out by means of an epithelial voltmeter (EVOM2, World Precision Instruments Inc., Hertfordshire, UK), following the experimental procedure previously described by the authors (Movia et al., 2018). To minimize reading errors, measurements were repeated three times for each sample.

## Statistical analysis

Graph-Pad Prism 8 (Graph-Pad Software Inc., La Jolla, CA, USA) was used to carry out the statistical analysis. A p value < 0.05 was considered statistically significant. The statistical tests used are indicated in the corresponding figure caption.

# Supplementary References

Di Cristo, L., Maguire, C.M., Mc Quillan, K., Aleardi, M., Volkov, Y., Movia, D., and Prina-Mello, A. (2018). Towards the Identification of an In Vitro Tool for Assessing the Biological Behavior of Aerosol Supplied Nanomaterials. *Int J Environ Res Public Health* 15.

Movia, D., Bazou, D., and Prina-Mello, A. (2019). ALI multilayered co-cultures mimic biochemical mechanisms of the cancer cell-fibroblast cross-talk involved in NSCLC MultiDrug Resistance. *BMC Cancer* 19**,** 854.

Movia, D., Bazou, D., Volkov, Y., and Prina-Mello, A. (2018). Multilayered Cultures of NSCLC cells grown at the Air-Liquid Interface allow the efficacy testing of inhaled anti-cancer drugs. *Sci Rep* 8**,** 12920.

Movia, D., Di Cristo, L., Alnemari, R., Mccarthy, J.E., Moustaoui, H., Lamy De La Chapelle, M., Spadavecchia, J., Volkov, Y., and Prina-Mello, A. (2017). The curious case of how mimicking physiological complexity in in vitro models of the human respiratory system influences the inflammatory responses. A preliminary study focused on gold nanoparticles. *Journal of Interdisciplinary Nanomedicine* 2**,** 110-130.
